# The Bull’s Eye Pattern of the Tear Film in Humans during Visual Fixation on En-Face Optical Coherence Tomography

**DOI:** 10.1038/s41598-018-38260-5

**Published:** 2019-02-05

**Authors:** Pietro Emanuele Napoli, Matteo Nioi, Ernesto d’Aloja, Maurizio Fossarello

**Affiliations:** 1 From the San Giovanni di Dio hospital, Clinica Oculistica, Azienda Ospedaliera Universitaria di Cagliari, Cagliari, Italy; 2 From the Department of Surgical Sciences, Eye Clinic, University of Cagliari, Cagliari, Italy; 3 From the Department of Medical Sciences and Public Health – Forensic Science Unit –University of Cagliari, Cagliari, Italy

## Abstract

The aim of the study was to define and characterize the optical behavior of the tear film during visual fixation in humans on en-face optical coherence tomography (OCT). We included 20 healthy participants, 60% female, aged from 25 to 42 years (33.05 ± 4.97 [mean ± SD]) and ten patients with severe dry eye, 50% female, aged from 26 to 42 years (33.7 ± 5.31). To perform high-resolution tear film imaging, participants were asked to gaze at the internal fixation point in the spectral-domain anterior segment OCT device, and meanwhile scanning session was executed at the following time-points after blinking: at the 2^nd^, 3^rd^, 4^th^, 5^th^, and 6^th^ second. After one hour, OCT imaging was repeated (*second session*) by a different operator masked to the study to verify the reliability of results. During each measuring session, a pulse oximetry was used for continuously measuring the heart rate and oxygen saturation (SpO_2_%). A preliminary experiment was also performed to test the absence of *geometric patterns* from the anterior surface of a *motionless* artificial eye. OCT imaging showed a motionless, stable anterior surface of the artificial eye and in dry eye patients. Conversely, in the healthy participants of the study, a bull’s eye pattern of the tear film was detected by OCT at the 2^nd^, 3^rd^, 4^th^, 5^th^, and 6^th^ second after blinking, respectively, in 45%, 60%, 45%, 60%, and 40% of OCT scans during the first session, and in 35%, 65%, 65%, 60%, and 35% of cases in the second session. Overall, a total of 200 OCT scans were performed in normal human population. A significant correlation was found between the novel tear film pattern and heart rate during the first and the second session (p < 0.01) in healthy eyes. Conversely, no correlation was revealed with SpO2%. Intraclass correlation (ICC) analysis for OCT imaging of the tear film revealed a statistically significant reproducibility of the results (ICC = 0.838; p < 0.01), indicating the high level of reliability of the method, independently of heart rate and SpO2% variables. There exists a novel, geometric pattern of the tear film during visual fixation detectable by en-face OCT, which is mainly evident as heart rate increases. Its discovery implies in turn the presence of a specific vibration (or imperceptible motion) of the tear film that, at present, is not recognized and corrected by the OCT software (in image postprocessing) unlike other eyeball movements.

## Introduction

The tear film is the predominant optical element of the eye diopter, with a refractive power of more than 43 diopters, and the first barrier against external agents, including the ultraviolet (UV) light rays^1–5^.

In the current consensus, the tear film *behavior* during visual fixation, at least in healthy subjects, is considered apparently quiescent, although *fixational eye movements* (i.e., microsaccades, ocular drifts, and ocular microtremor) normally occur^6,7^. Of note, the latter are not visible to the human eye or with the traditional eye tests, since their amplitudes vary approximately from 2 to 120 arcminutes^6^. Clearly, small eye movements may be also determined by *retinal artery pulsation*^8,9^.

From a theoretical point of view, these *continuous* movements of the eye may transfer energy to the ocular surface, thus resulting in a mechanical stimulation, repetitive and transient, of the tear film. Nevertheless, to our knowledge, no activity or propagation of energy of the tear film during visual fixation has yet been reported in the literature.

The purpose of our work was to study the en-face OCT optical behavior of the precorneal tear film during visual fixation. Moreover, we examined the correlation between the OCT findings and the heart rate or oxygen saturation, as well as the reliability of results.

## Methods

The study was conducted in accordance with the Declaration of Helsinki guidelines and approved by the local Office of Research Ethics (University of Cagliari). Written informed consent was obtained from all participants.

Twenty healthy subjects, 60% female, aged from 25 to 42 years (33.05 ± 4.97 [mean ± SD]), and ten patients with severe dry eye, 50% female, aged from 26 to 42 years (33.7 ± 5.31), were recruited from patients of the Eye Clinic (Department of Surgical Sciences, Cagliari, Italy) and volunteers from the School of Ophthalmology (University of Cagliari, School of Ophthalmology).

As previously reported, all subjects underwent a standard clinical evaluation that included a complete history and a detailed ocular surface examination^10–12^. The latter consisted of a series of tests carried out in the following order: a standard questionnaire for dry eye (McMonnies questionnaire), fluorescein tear break-up time (FTBUT), fluorescein staining of the ocular surface (Oxford system), the Schirmer secretion test without anesthesia, and a biomicroscopic examination of the meibomian glands and lid margins. Based on the results of these tests, the inclusion criteria for healthy eyes were: no significant symptoms of ocular irritation (OSDI < 12), FTBUT > 10 seconds, Oxford scheme ≤ panel A, and Schirmer 1 test score of more than 10 mm in 5 minutes.

Subjects with any systemic disease, ocular surface diseases occurred during the past six months (including the meibomian gland dysfunction), any evidence of lid abnormality or abnormal blinking, systemic or topical medication, history of eye surgery or contact lenses wear, were excluded from the study.

The various steps of the study were performed under the same conditions of humidity (within a range of 50–60%) and temperature (within a range of 12–22 °C) in a dimly lit room, as previously described^13,14^.

### En-face OCT imaging

The OCT imaging was performed by means of a Cirrus HD-OCT 5000 device. This spectral-domain OCT platform (Carl Zeiss Meditec Inc, Dublin, CA, USA) uses a wavelength of 840 nm, takes 68,000 axial scans per second, and has a 5-µm axial resolution (in tissue). Moreover, a tracking mechanism is also available in order to trace and compensate/reduce eye motion artifacts in real time.

The Anterior Segment scanning protocol (Cube 512 × 128) was used to acquire all the tear film images. This approach acquires a series of 128 horizontal scan lines each composed of 512 A-scan, thus generating a volume of data through a 4 mm square grid. It also acquires a pair of high definition scans (i.e. lines of 1024 A-scans) through the center of the cube in the vertical and horizontal direction. This scanning protocol therefore creates a 3-D image of the data.

During each scanning section, patients were instructed to gaze at the central target in the OCT device, and were asked to hold the breath to avoid any movement of the head. In the meantime, the visual fixation was constantly monitored, so that if the patient moved the eyes (or the head), the OCT scan was repeated.

Since the tear film is normally spread in ~ 0.5 s over the whole ocular surface and re-compressed close to eyelid margins approximately every 5 s after each blink (or alternatively it remains intact before break up every 7–10 s in healthy patients), we performed the OCT scans one by one at the following time-points after blinking (*first session*): at the 2^nd^, 3^rd^, 4^th^, 5^th^, and 6^th^ second^15,16^. Before each scan (for each time-point), patients closed their eyes and opened again them without blinking, until the individual image was acquired. After one hour, OCT imaging was repeated (*second session*) by a different operator masked to the initial results to verify the data reliability, and therefore to exclude the possibility of an optical effect due to random variables (during the sampling or the reconstruction stage).

During each measuring session, a pulse oximetry was used for continuously measuring the heart rate (pulse) and the oxygen saturation (SpO_2_%). The heart rate was considered as an indirect measure of the retinal artery pulsation. SpO_2_% was monitored to exclude hypoxemia (e.g. caused by hypoventilation) in each imaging session.

OCT scans were considered *eligible* for our analysis based on the following conditions: absence of *excessive* artifacts owning to eyelid margin, pulse within a range of 60–100 beats/min, SpO_2_% ≥95%. Otherwise, results were excluded and OCT imaging repeated.

### Tear film Patterns on en-face OCT imaging

Two main appearances of the tear film surface were detected by en-face OCT:

1. The *bull’s eye pattern* was identified by the presence of concentric rings, well distinguishable from each other by the different reflectivity, that periodically reverse their intensity (with high and low color scale values) from the center towards the periphery. This *geometric pattern* (as a representation of various figures) was characterized by an inhomogeneous distribution of OCT signal with irregular reflectivity on entire scanned area (Fig. 1).

**Figure 1:**
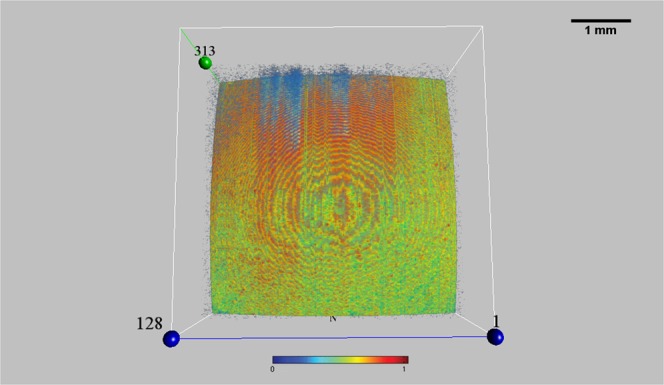
A Bull’s Eye Pattern from the tear film surface on en-face OCT imaging. This *geometric pattern* was characterized by multiple figures, mainly concentric circles, which cyclically reverse their intensity (with different color scale values) from the center towards the periphery. This bull’s eye appearance was detected only in healthy subjects, and never in the context of the cornea (see also Video SI2). (Sectors were as follows: N: nasal; T: temporal; S: superior; I: inferior. Each scan line within the data volume was labeled with a number. Scale bar = 1 mm). Reflectivity of the en-face image [Color bar: intensity = 0 (dark blue) to intensity = 1 (red)].
2. In contrast, en-face OCT images free from concentric circles (Fig. 2) were characterized by a reflectivity approximately homogeneous (except for some lines) over the entire scanned area (that we defined as *regular*, stable, or quiescent pattern).

**Figure 2:**
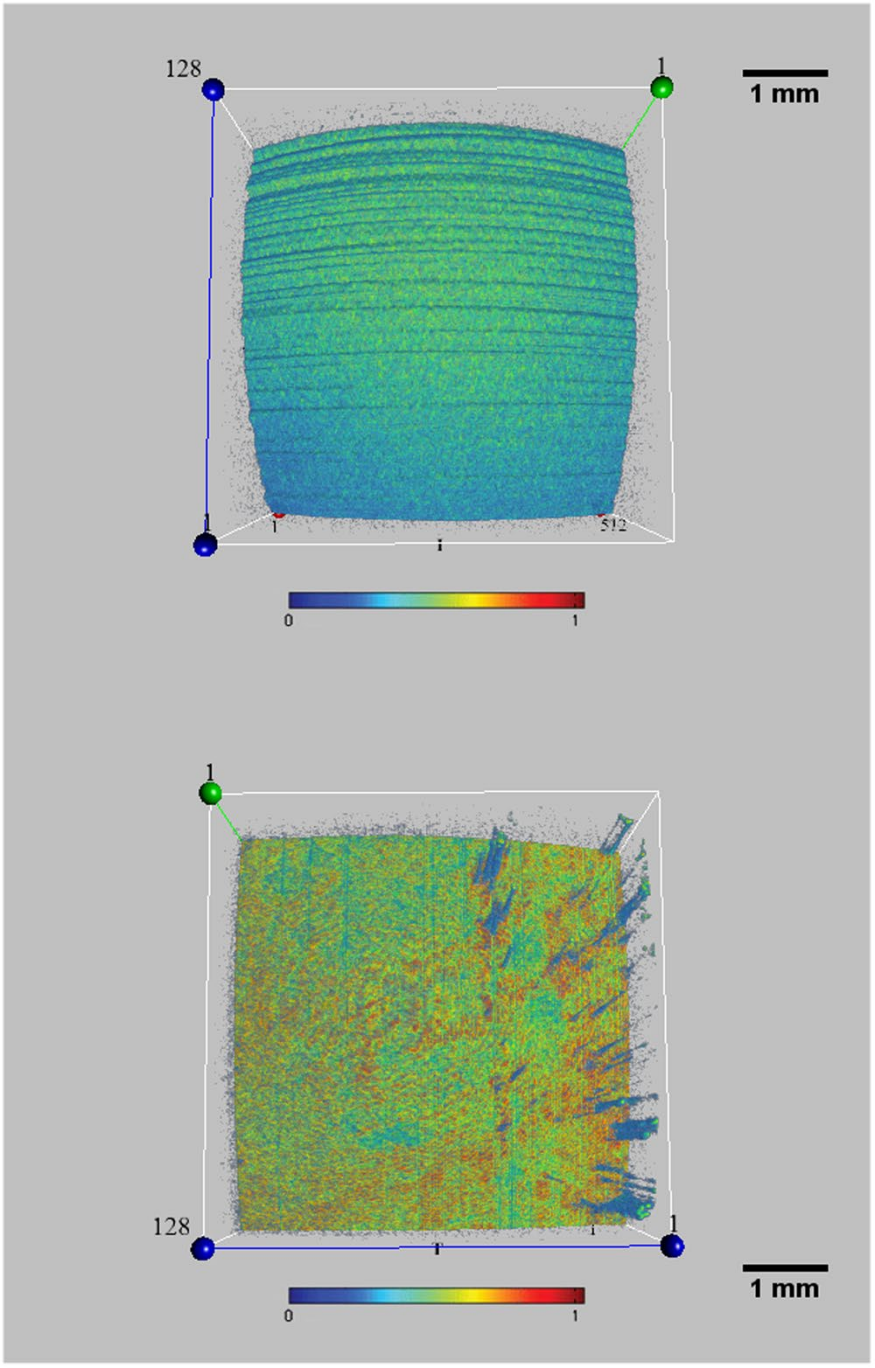
A regular, tear film pattern (free from geometric figures) by en-face OCT imaging. A reflectivity approximately homogeneous (except for some lines, or corneal reflex artifacts) over the tear film surface was observed in OCT images free from concentric circles. We defined this pattern as *regular*, stable, or quiescent. This appearance was found both in the artificial eye (top), and in all dry eye patients (bottom). (Sectors were as follows: N: nasal; T: temporal; S: superior; I: inferior. Each scan line within the data volume was labeled with a number. Scale bar = 1 mm). Color bar as in Fig. 1.

### Preliminary experiment n. 1

#### (Motionless, Artificial eye)

**Figure 3:**
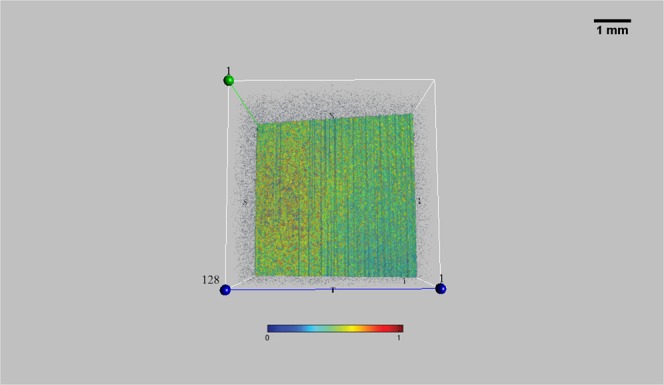
En-face OCT imaging of an artificial eye, motionless and dry. En-face OCT scan of an artificial eye. To verify the presence of *patterns of geometric figures* from the anterior surface of the eye (see text), a “test eye” (normally used for calibration of IOLMaster biometer) was placed on a mechanical support in front of OCT device. A stable anterior surface of the artificial eye was obtained by OCT imaging, confirming the absence of geometric figures or image distortion (please see also Video SI1). (Sectors were as follows: N: nasal; T: temporal; S: superior; I: inferior. Each scan line within the data volume was labeled with a number. Scale bar = 1 mm). Color bar as in Fig. 1.

A preliminary experiment was performed to test the absence of *geometric patterns* from the anterior surface of a *motionless* artificial eye. For this purpose, a “test eye”, normally used for calibration of IOLMaster biometer (Carl Zeiss Meditec), was placed on a mechanical support in front of OCT device. Radius of curvature of the “test eye” was 7.18 ± 0.03 mm. As a result, OCT imaging showed a motionless, stabile anterior surface of the “test eye” (Fig. 3; Video SI1).

**Figure 4:**
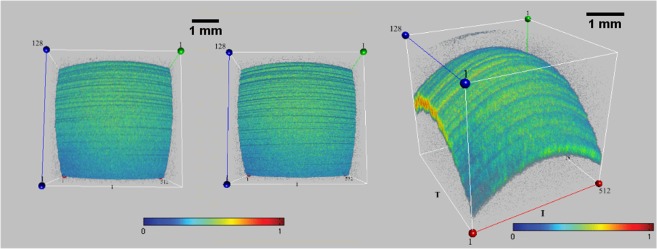
En-face OCT imaging of an artificial eye with a mucomimetic or lipid layer. A mucomimetic (left) and a lipid-based (middle) artificial tear was separately instilled on two artificial eyes in order to unveil the optical behavior of the *mucoaqueous* and *lipid* layer on en-face OCT imaging (e.g. verifying the presence of a hypothetical *thin-film interference* effect). As a result, a regular pattern with increased reflectivity was detected in both cases, but the bull’s eye appearance has never been detected. The 3-D image of the artificial tear film (right) helps to understand the great ability to reflect the OCT signal from these layers. (Sectors were as follows: N: nasal; T: temporal; S: superior; I: inferior. Each scan line within the data volume was labeled with a number. Scale bar = 1 mm). Color bar as in Fig. 1.

Furthermore, to investigate the potential role of the mucoaqueous and lipid layer on the ocular surface in OCT signal transmission (e.g. verifying a hypothetical *thin-film interference* effect), we separately instilled a calibrated quantity (35 µl, with a micropipette) of a mucomimetic (MAT) and of a lipid-based (LAT) artificial tear. Surprisingly, a more homogeneous reflectivity was detected in both cases, indicating the great ability to reflect the OCT signal from the wet surface of the eye, but the “bull’s eye pattern” has never been detected (Fig. 4).

### Preliminary experiment n. 2

#### (Dry Eyes)

In order to unveil the OCT optical behavior of human eyes in absence (or paucity) of tears, we performed the scanning protocol also in ten patients with severe dry eye syndrome (whose diagnosis was established as reported in previous studies)^11,12^.

This allowed us to understand the different role of the tear film and of fixational eye movements in OCT signal transmission, separately. In fact, the bull’s-eye pattern was observed in no patient with dry eye, thus demonstrating that a dry ocular surface in humans, despite the fixational movements, is not sufficient to determine a geometric pattern (with inhomogeneous appearance) of the tear film on OCT imaging.

This in turn means that, as with posterior segment imaging, many irregularities of the anterior segment are normally corrected by OCT software, but this is possible as long as there is little (or no) liquid in motion above the tissue being scanned. Indeed, a liquid may have continuous deformations (since a fluid represents a state of matter with no fixed shape) which can be difficult to “attenuate” or “smooth” successfully in image postprocessing.

### Statistical analysis

Statistical analyses were executed via computer with a statistical software package SPSS for Windows version 21.0 (SPSS Inc., Chicago, Illinois). The normality of data distributions was performed by Shapiro-Wilk test and Kolmogorov-Smirnov test with Lilliefors correction.

Correlation analysis between OCT results (i.e. the number of times the oscillations of the tear film were detected for session) and heart rate (pulse) or oxygen saturation (SpO_2_%) was determined by calculating Spearman’s ρ test and Kendall’s τ test. The OCT data obtained at the second scanning session were used for the reproducibility analysis. Accordingly, the correlation between measurements was evaluated by the intraclass correlation coefficient (ICC).

The sample size calculation was performed accepting an alpha risk of 0.05 and a beta risk of 0.2 in a two-sided test. To recognize as statistical significant a correlation coefficient greater than or equal to 0.86 would require eight patients.

Statistical significance was defined as *P* < 0.05.

## Results

The results of instrumental and clinical exams (pulse and SpO2%) are reported in Table 1–3. The heart rate was 74.7 ± 10.15 beats/min, and SpO2% was 98.4 ± 0.99%. Informative images of tear film behavior were obtained in all participants.

**Table 1:** Detection of the bull’s eye pattern of the tear film by OCT in healthy subjects.

	Scanning time after blinking (seconds)				
	2^nd^	3^rd^	4^th^	5^th^	6^th^
Scanning session 1*	45% (9/20)	60% (12/20)	45% (9/20)	60% (12/20)	40% (8/20)
Scanning session 2*	35% (7/20)	65% (13/20)	65% (13/20)	60% (12/20)	35% (7/20)

^*^Absolute frequencies and percentages.

OCT scans were performed at the following time-points after blinking (*first session*): at the 2^nd^, 3^rd^, 4^th^, 5^th^, and 6^th^ second. Before each scan (for each time-point), patients closed their eyes and opened again them without blinking, until the individual image was acquired. After one hour, OCT imaging was repeated (*second session*) by one different operator masked to the study to verify the reliability of results. Thus, the bull’s eye pattern was detected in 50% (50/100) and in 52% (52/100) of OCT scans during the session 1 and 2, respectively.

**Table 2:** Individual OCT findings, heart rate and oxygen saturation (SpO_2_) in healthy subjects.

	Scanning session 1*	Scanning session 2*	Heart rate (beats/min)	SpO_2_ (%)
Patient n. 1	0	0	60	99
Patient n. 2	1	2	63	99
Patient n. 3	3	3	80	98
Patient n. 4	3	3	77	98
Patient n. 5	4	5	84	98
Patient n. 6	4	4	85	98
Patient n. 7	2	2	70	99
Patient n. 8	2	2	72	99
Patient n. 9	0	0	64	97
Patient n. 10	0	0	61	96
Patient n. 11	1	2	67	99
Patient n. 12	4	4	82	99
Patient n. 13	4	4	84	98
Patient n. 14	5	5	90	99
Patient n. 15	2	3	70	100
Patient n. 16	2	2	70	100
Patient n. 17	3	1	75	98
Patient n. 18	4	4	88	97
Patient n. 19	1	1	62	99
Patient n. 20	5	5	90	98

^*^Number of times the bull’s eye pattern was detected (or imaged).

**Table 3:** Correlation Coefficients between OCT results and heart rate or oxygen saturation (SpO_2_), and reliability results in healthy subjects.

		Heart rate (beats/min)	SpO_2_ (%)
*Scanning session 1*	Spearman’s ρ test	ρ_1_ = 0.979, (p < 0.01)	ρ_1_ = −0.194 (p = 0.41)
	Kendall’s τ test	τ_1_ = 0.924, (p < 0.01)	τ_1_ = −0.178 (p = 0.33)
*Scanning session 2*	Spearman’s ρ test	ρ_2_ = 0.943, (p < 0.01)	ρ_2_ = −0.13 (p = 0.58)
	Kendall’s τ test	τ_2_ = 0.86, (p < 0.01)	τ_2_ = −0.128 (p = 0.50)
Reproducibility^a^	Intraclass correlation^b^ = 0.721 (p < 0.01)		
	Intraclass correlation^c^ = 0.838 (p < 0.01)		

Reproducibility: Two-way mixed effects model where people effects are random and measures effects are fixed.

^a^Type C intraclass correlation coefficients using a consistency definition-the between-measure variance are excluded from the denominator variance.

^b^The estimator is the same, whether the interaction effect is present or not.

^c^This estimate is computed assuming the interaction effect is absent, because it is not estimable otherwise.

Overall, a total of 200 OCT scans were performed in healthy subjects. Thus, the bull’s eye pattern was found in 50% (50/100) and 52% (52/100) of OCT images during the session 1 and 2, respectively. Specifically, it was detected at the 2^nd^, 3^rd^, 4^th^, 5^th^, and 6^th^ second after blinking, respectively, in 45%, 60%, 45%, 60%, and 40% of cases during the first session, and in 35%, 65%, 65%, 60%, and 35% of scans in the second session (see Fig. 1; Table 1; Video SI2).

A significant correlation was found between the tear film pattern and the heart rate during the first (τ_1_ = 0.924, p < 0.01; ρ_1_ = 0.979, p < 0.01) and the second session (τ_2_ = 0.86, p < 0.01; ρ_2_ = 0.943, p < 0.01). Conversely, no correlation was revealed between the tear film pattern and the SpO2% (τ_1_ = −0.178; ρ_1_ = −0.194; τ_2_ = −0.128; ρ_2_ = −0.13).

Reproducibility analysis demonstrated a significant coefficient of intraclass correlation (ICC) value for OCT imaging of tear film pattern (ICC = 0.838; p < 0.01), indicating the high level of reliability of our results, independently of pulse and SpO2% variables.

With regard to the patients with dry eye and to the artificial eye, the bull’s eye pattern was not detected under any circumstances.

## Discussion

In the present work, we revealed for the first time the existence of a novel, bull’s eye pattern of the tear-film free-surface on OCT imaging during visual fixation, i.e. in a condition in which the eyes are only apparently motionless. In addition, our preliminary experiments clearly demonstrated that this geometric pattern cannot occur in absence (or paucity) of tears or when small movements of eyes (and tears) are missing. Consequently, this discovery implies in turn the *indirect* demonstration of the presence of slight movements of the precorneal tear film during visual fixation, which are not necessarily aligned (or *synchronous*) with the movements of the cornea (e.g. during the pulsation of the retinal artery) (see Video SI2).

As in case of the *wagon-wheel effect*, the movements of the object being scanned may lead to an *aliasing* effect in OCT imaging with the appearance of a novel, particular pattern^17^. In our study, the bull’s eye pattern was specific at the level of tear film and missing in the rest of the cornea.

Generally, aliasing effects (e.g. wagon-wheel effect) occur when there is an unfit sampling of the signal, which results in a production of the reconstructed signal with different characteristics from the original ones (aliases), or between overlapping transparent objects (*moiré effect*)^18^.

In accordance with this, the bull’s eye pattern was observed only in healthy subjects, i.e. in case of a normal tear film, and therefore in presence both of tears and of physiological small eye movements. Conversely, as demonstrated in an artificial eye and in patients with dry eye, a motionless surface or a dry eye state cannot cause a similar *artifact*.

Therefore, although OCT software can successfully attenuate some irregularities or distortions, as it happens regularly for posterior segment imaging, this procedure can be adequately performed if there is little (or no) liquid in motion above the tissue being scanned. In fact, a liquid as the tear film is easily deformable (i.e. changeable in shape, but not in volume) and it may have continuous deformations, which may be difficult to correct successfully in image postprocessing.

As demonstrated in an experimental study, a Newton’s rings fringe pattern (a similar interference pattern) can be created based on the position of the incident beam on the galvoscanner mirror and on the interface optics used, so that the reference-arm length is thought of as the equivalent of the distance to surface under test^19,20^. Clearly, a vibrating tear film (above a curved, ocular surface) may also create a variable, optical path length difference of reflected rays from its moving surface.

As is known, perfect immobility is alien to biological systems. Likewise, when fixating a target, the eyes of both human and non-human primates are known to perform small movements, called *fixational eye movements*, whose role has been broadly discussed and debated for a long time^6^. In the current consensus, fixational eye movements contribute to *maintaining visibility*, by continuously stimulating neurons in the early visual area of the brain, which mostly respond to transient stimuli. In the absence of retinal jitter (a laboratory condition called retinal stabilization), stabilized images as a visual percept rapidly fade out and completely disappear^21^. On the other hand, retinal artery pulsation may also contribute to determine small eye movements on the antero-posterior axis^8^.

In general, artifacts are considered negative features in OCT imaging that is desirable to correct (or attenuate), but in some cases they can lead to the understanding of novel aspects of ocular physiology, as happened in the present study^22–25^. Indeed, the bull’s eye pattern of the tear-film free-surface reveals the existence of a *motion* of the tear film (in addition to those already recognized and corrected by the OCT software), which can be detected in absence of other deformations in the cornea, probably due to the inertial forces of the liquid mass (i.e. the tear film) with respect to the solid mass (i.e. the ocular surface). As a consequence, this finding implies in turn the presence of a specific *vibration* of the precorneal tear film beyond the well-known movements of the ocular structures that are regularly hidden by image reconstruction techniques (in image postprocessing).

Theoretically, in the absence of fixational eye movements and other tremors, the free surface of a physiological tear film would be homogeneous and free from undulations. On the contrary, the presence of a bull’s eye pattern implies a transfer of energy derived from small movements of the ocular surface to the human tears, similarly to what happens in the free surface of a liquid that vibrates inside a moving vessel.

Although the exact movement of the tear film is currently difficult to be imaged, it is plausible that it is very similar to a wave motion, roughly alike to the bull’s eye OCT pattern (in which, concentric circles might refer to some wavefronts of human tears). In fact, as is known, if the free surface of a liquid is stimulated, waves are elicited on the surface.

This previously unknown vibration of normal human tears clearly may have a variable number of implications, both *optical* and *biological*, and suggests a novel, theoretical model of the tear film non-quiescent, in continuous motion.

**Figure 5:**
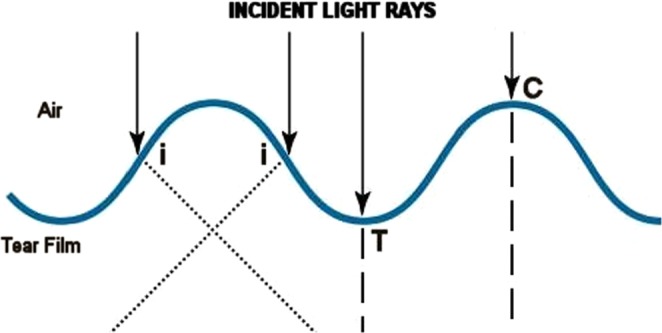
Schematic diagram of coordinate system (x, y) used to describe the interaction between a wavy tear film and light rays. Propagation of light rays (*arrows*) at the interface between air and tear film depends on whether they travel *perpendicular* to the boundary between the two media (angle of incidence measured from the normal, θ_i_ = 0) or with an oblique angle (θ_i_ ≠ 0). Exact values of refractive indices are assumed to be non influential, for simplicity. Considering the light rays *perpendicular* to the tear film plane, the movements of the tears, acting as “irregularly-curved surfaces” or irregular lenses (i.e. as an inhomogeneous medium), can lead to changes in direction of propagation of incident light rays depending on whether they strike a point of surface wave “intermediate” (*i*), or “extreme” (that is the *crest* (C) or the *trough* (T), which are respectively the highest and the lowest point of the disturbance in the tear film). In particular, at intermediate points (*i*) between C and T, there is a change in direction of incident light rays (since the refractive indices of the two media are different) that implies a deviation angle (θ_d_) in their propagation (*dotted lines*): θ_d_ ≠ 0. Conversely, the light rays that strike the most extreme points of the wave (traveling perpendicular to the boundary between the media) can continue their route beyond the free surface without being deflected (θ_d_ = 0), with a change only in their speed (*dashed lines*). Since the waves *travel* or *vibrate* through the free surface of the tear film, the propagation angle (θ_d_) of incident light rays varies over time and the spatial points placed behind the tear film are alternatively struck by them one after the other, cyclically.

From an optical point of view, the small movements of the tear film, acting as “irregularly-curved surfaces” or irregular lenses, can lead to cyclic changes in direction of propagation of incident light rays (see Fig. 5)^26,27^. This is of particular interest as regards the cyclical and transient stimulation of photoreceptors (i.e., for maintenance of vision), as well as the *photochemical*, or *light-driven, reactions* initiated/maintained by light radiation^21,28^. For this reason, all chronic movements of the tear film may have important implications on the eye physiology and the pathogenesis of various eye disorders (e.g., photo-oxidation related modifications)^4^. Consequently, we strongly believe that behavior abnormalities of the tear-film free-surface may lead to a loss of protection from UV light rays, and therefore predispose to some diseases of the ocular surface and cornea (e.g., photoconjuntivitis, photokeratitis, epithelial damage, altered natural cross-linking), lens pathologies (e.g., nuclear or cortical cataracts), glaucoma, vitreous abnormalities (e.g., topographically different metabolic areas), and retina/optic disc diseases (e.g., age-related macular degeneration, melanoma)^4,28–30^. Again, an *excessive* vibration the tear film, as might occur in patients with nystagmus, may lead to a *chaotic transmission* of light to the retina, thus resulting in visual impairment for young patients^31^.

On the other hand, the waves of the tear film may exert a *mechanical action* on the limbal stem cells (i.e., acting as a mild and physiological stress), thus determining the activation of the ATR protein, which is a protein stimulated by vibration^32^. By activation of the ATR protein, the tremors of the tear film may have a protective role for the limbal stem cells, which are normally exposed to UV light (e.g., sunlight), and a promoting action of the healing process at the level of the ocular surface^33^. Accordingly, the tumors and the altered competence of regenerating ocular surface epithelium might have a relationship with an abnormal behavior of the tear film (e.g., in dry eye patients)^32^.

Of note, our results show that the ability of OCT to image the bull’s eye pattern of the tear film increases as the heart rate rises. This finding implies the existence of a *relationship* among the lacrimal system, the vascular system (heart rate), and the neuroscience (fixational eye movements), thus suggesting a possible relationship between various systemic medications (e.g. drugs acting on the cardiovascular system or on the nervous system) and the ocular surface (e.g., dry eye).

Moreover, it is possible to hypothesize that OCT imaging of the bull’s eye pattern was easier to perform in the *short instants* in which the waves generated by the retinal artery pulsation were overlapped to those due to fixational movements (*constructive interference*).

Similarly to the rapid eye movements (R.E.M) that obviously take place during eyelid closure (sleeping and dreaming), one of the functions of the slight movements of the tear film during eye fixation may be the *mixing* of the tear film, that is to say of nutrients and waste substances during the period of eye opening^34,35^.

Although some interference patterns are generally due to the overlap of transparent layers (such as the *moiré effect*), at the moment we cannot be sure about which specific tear layer (or interaction between tear layers) can give rise the bull’s eye pattern, since we have disclosed, in a motionless artificial eye, that neither the aqueous nor the lipid component of the tear film is by itself able to determine it^36^.

Our study has some limitations. Since the normal geometry of the cornea is characterized by a central protrusion, which potentially explains the detection of the pattern mainly at the central level, it would be desirable to study various corneal geometries (e.g. keratoconus). Moreover, these observations should be compared and validated with different OCT technologies (e.g. swept source, full-field, or micro-OCT approaches)^37,38^. Clearly, potential age- and gender-related variations should be further evaluated with a more comprehensive sample of healthy subjects. Again, future studies would be used to evaluate the effect of systemic drugs associated with dry eye (e. g., psychotropic drugs, or drugs for hypertension) or the role of several diseases on the modulation of the bull’s eye pattern of the tear film.

In conclusion, there exists a novel, bull’s eye pattern of the tear film during visual fixation detectable by en-face OCT, which is mainly evident as heart rate increases. Its discovery implies in turn the presence of a specific vibration of the tear film that, at present, is not recognized and corrected by the OCT software (in image postprocessing) unlike other eyeball movements. Moreover, this finding may have an important, promoting role in the understanding of physiology of vision and of various ocular diseases (e.g., dry eye, nystagmus and light-related diseases), and in the evaluation of relationship among the lacrimal system, the vascular system (heart rate), and the nervous system (fixational movements).

## Supplementary information

LEGENDS FOR SUPPORTING MOVIES

Three-dimensional OCT imaging of an artificial eye, motionless and dry (Black-and-white video).

Three-dimensional OCT imaging of cornea from the anterior to posterior surface.

## Data Availability

All data are reported in the present manuscript.
