# Supplementary material for: The Bull’s Eye Pattern of the Tear Film in Humans during Visual Fixation on En-Face Optical Coherence Tomography
Source: Sci Rep. 2019 Feb 5;9:1413. doi: 10.1038/s41598-018-38260-5 (PMC6363734; doi:10.1038/s41598-018-38260-5)
Supplement: Supplementary file 1 — LEGENDS FOR SUPPORTING MOVIES [file 41598_2018_38260_MOESM1_ESM.pdf]

LEGENDS FOR SUPPORTING MOVIES

Title of the manuscript: **The Bull's Eye Pattern of the Tear Film in Humans during Visual Fixation on En-Face Optical Coherence Tomography.**

Authors list: Pietro Emanuele Napoli, Matteo Nioi, Ernesto d'Aloja, Maurizio Fossarello.

Scientific Journal: Scientific Reports

VIDEO 1. Three-dimensional OCT imaging of an artificial eye, motionless and dry (Black-and-white)**.** A stabile anterior surface of the artificial eye was obtained by OCT imaging, confirming the absence of geometric figures or image distortion. This preliminary experiment was performed to test the absence of *geometric artifacts* (see text) from the anterior surface of a "test eye", which was placed on a mechanical support in front of OCT device. (Sectors were as follows: N: nasal; T: temporal; S: superior; I: inferior. Each scan line within the data volume was labeled with a number).

VIDEO 2. Three-dimensional OCT imaging of cornea from the anterior to posterior surface. The geometric pattern, characterized by multiple concentric circles, was *well-evident* on the tear film surface (which is in a state of matter easily changeable in shape) but not in the context of the corneal tissue (see text). Hence, the bull's eye appearance was detected in absence of other deformations of the cornea, probably due to the inertial forces of the liquid mass (i.e. the tear film) with respect to the solid mass (e.g. the ocular surface). (Sectors were as follows: N: nasal; T: temporal; S: superior; I: inferior. Each scan line within the data volume was labeled with a number). Color bar as in fig. 1.
